# Associations between urinary concentrations of bisphenols and serum concentrations of sex hormones among US. Males

**DOI:** 10.1186/s12940-022-00949-6

**Published:** 2022-12-22

**Authors:** Chichen Zhang, Yuehong Luo, Shi Qiu, Xinyi Huang, Kun Jin, Jiakun Li, Mi Yang, Dan Hu, Xiaonan Zheng, Zhongyuan Jiang, Mingda Wang, Xiaoli Zou, Qiang Wei

**Affiliations:** 1 Department of Urology, Institute of Urology, West China Hospital of Sichuan University, Chengdu, 610041 Sichuan Province China; 2 Department of Anesthesia, First Affiliated Hospital, School of Medicine, Zhejiang University, Zhejiang, 310009 Hangzhou China; 3 Department of Sanitary Technology, West China School of Public Health and West China Fourth Hospital, Sichuan University, Chengdu, China; 4 Department of Clinical Research Management, West China Hospital of Sichuan University, Chengdu, 610041 China

**Keywords:** Sex hormones, Bisphenol A, Bisphenol F, Bisphenol S

## Abstract

**Background:**

Bisphenol A (BPA) exposure and its structural analogs (BPS and BPF) might cause endocrine alterations and adverse physiological effects. Few studies to date have directly explored the association between its structural analogs (BPS, BPF) and sex hormones in adult male participants. Therefore, we aimed to assess the associations between BPA, BPS, BPF, and sex hormones in American adult men.

**Methods:**

We used data from the U.S. National Health and Nutrition Examination Survey 2011–2016. We excluded participants without data available on sex hormones and urinary bisphenols. Furthermore, participants consuming sex hormone medications were excluded. Multivariable regression models were performed to assess the association between bisphenols and sex hormones.

**Results:**

In this study, 2367 participants were included. Of 2367, in 1575 participants, the data on BPS and BPF were available. We found that a per unit increase in BPF was associated with 0.575 ng/dL higher total testosterone (TT) (Model 2: 95% CI: 0.047, 1.103, *P* = 0.033). However, there was no significant association between BPA or BPS and TT. Furthermore, increased BPA and BPS levels were associated with higher levels of sex hormone-binding globulin (SHBG) (Model 2: β = 0.364, 95% CI: 0.158, 0.571; β = 0.25, 95% CI: 0.071, 0.429, respectively). Additionally, participants in the highest BPA exposure quartile (quartile 4) had 4.072 nmol/L higher levels of SHBG than those in quartile 1 (Model 2: 95% CI: 0.746, 7.397, *P* = 0.017; P for trend =0.005). Both BPA and BPS were negatively associated with free testosterone (FT, nmol/L) after full adjustment (Model 2, β = − 0.01%, *P* = 0.0211, P = 0.0211; Model 2, β = − 0.01%, *P* = 0.0258, respectively). However, BPF was positively associated with FT (Model 2, β = 0.0029%, *P* = 0.0028).

**Conclusion:**

Our study indicated that exposure to both BPA and its substitutions could alter sex hormone levels. This finding supports the possibility that human exposure to bisphenols at environmental levels might affect the endogenous hormone balance.

**Supplementary Information:**

The online version contains supplementary material available at 10.1186/s12940-022-00949-6.

## Introduction

Sex hormones are critical in numerous biological processes, including reproduction, differentiation, development, inflammation, and metabolism [1]. The role of testosterone (T), the principal androgenic hormone, is important in human anabolism. It is essential for endocrine functions and spermatogenesis of the male testis. The majority of the circulating testosterone in blood is commonly bound to sex hormone-binding globulin [SHBG] and albumin [2]. Estradiol (E_2_) is essential for female reproductive functions. Estrogen hormone decline is associated with a higher risk of cognitive impairment and cardiovascular disease [3]. Moreover, the liver produces SHBG and secretes it into the blood. SHBG, the transport protein, binds to testosterone and estradiol and thus regulates their bioavailability [4]. Environmental insults impair human health around the world; sex hormones are reportedly vulnerable to environmental and chemical exposure, especially endocrine-disrupting chemicals (EDCs) [5].

Bisphenol A (BPA), characterized as an EDC [6], is widely produced primarily for manufacturing polycarbonate plastics (e.g., baby bottles, sports equipment, and dental sealants) [7] and epoxy resins [8] (e.g., food and beverage containers). In human, the major route of BPA exposure is food or beverage ingestion contaminated with leached BPA from the packaging material [9]; other routes include skin contact and indoor dust inhalation [10]. It has been established that BPA can bind to estrogen receptors and androgen receptors. Thus, it can affect steroidogenic enzyme expression and interfere with sex steroid levels [11]. Numerous animal studies have demonstrated that exposure to BPA has adverse reproductive and behavioral outcomes [12–14]. Similarly, emerging human researches suggest that BPA has unfavorable impacts on the reproductive system of both sexes, metabolic processes, like obesity and diabetes, and cardiovascular diseases [15, 16]. Therefore, safety concerns related to BPA exposure have resulted in the gradual substitution of BPA with its alternatives, including bisphenol S (BPS) and bisphenol F (BPF), in products advertised as “BPA free” [17–19].

Currently, BPF and BPS have also been identified with estrogenic and anti-androgenic features because these analogs have similar chemical structures to BPA [20]. Additionally, as per in vitro and in vivo studies, they cause similar endocrine alterations and adverse physiological effects [14, 21, 22]. Recently, Wang et al. focused on associations of urinary bisphenol concentrations with adverse effects on sex hormones in children and adolescents, revealing that BPA analogs (BPS and BPF) could have comparable endocrine disrupting effects as those possessed by BPA [23]. However, literature exploring the impact of bisphenol A exposure on sex hormones homeostasis is scanty with contradictory data [24, 25]. Pollock et al. used data from the Canadian and U.S. populations and demonstrated that no association between BPA and total T (TT) or E_2_ was found in male participants, but they did not assess the relationship between BPA and other sex hormone indicators (e.g., SHBG and calculated free T). Lassen et al. showed that increased urinary BPA levels were related to higher TT and were not significantly associated with SHBG in young Danish men [26]. To date, few studies have directly explored the association of BPA’s structural analogs with sex hormone levels in adult men [27].

Therefore, we aimed to explore the association of BPA and its analogs (BPS, BPF) with sex hormone levels in a nationally representative population of adult men.

## Methods

### Study population

The National Health and Nutrition Examination Survey (NHANES) is a continuous, national program that aims to evaluate the nutritional and health status of the U.S. population since 1999. The survey was conducted by the Center for Disease Control and Prevention (CDC) combining interviews and physical examinations. (Official website: http://www.cdc.gov/nchs/nhanes.htm). The Ethics Review Board (Continuation of Protocol #2011–17, National Center for Health Statistics) approved all procedures. Written informed consent was signed by all participants.

In this study, three-cycle data (2011–2012, 2013–2014, and 2015–2016) were extracted from the NHANES. Participants aged ≥20 years with data available on sex hormones, BPA, and its analogs (BPS, BPF) were included. Participants taking medication, including testosterone, estrogen, progesterone, or “other sex hormones (e.g., gonadotropin, sex hormone combination)” noted in the NHANES were excluded [28, 29]. Finally, 2367 participants were included. Of these, in 1575 participants, the data on BPS and BPF were available (NHANES 2013–2016). (Supplementary Fig. 1).

### Exposure assessments

Details on the laboratory methods and procedures of BPA, BPS, and BPF have been published previously [30]. Urinary BPF, BPS, and BPA were measured using solid phase extraction coupled online to high-performance liquid chromatography and tandem mass spectrometry. The lower limit of detection (LOD) was 0.2 μg/L for BPA and BPF and 0.1 μg/L for BPS from the NHANES website. Urinary BPA, BPS, and BPF concentrations below the lower LOD were substituted by LOD/ $\sqrt{2}$ based on the NHANES analysis guideline.

### Sex steroid measures

The testosterone, estradiol, and SHBG assessment methodologies have been reported elsewhere [31]. The LODs of TT, E2, and SHBG were defined as 0.75 ng/mL, 2.994 pg/ml, and 0.800 nmol/L, respectively. Sex hormones level below LOD were replaced by the LOD/ $\sqrt{2}$. In addition, free testosterone (FT, nmol/L) was calculated utilizing testosterone, albumin, and SHBG values [32]; the TT to E_2_ ratio (TT/E_2_) was employed to evaluate aromatase activity indirectly, which promoted the conversion of T to E_2_ [33].

### Covariates

As per previous studies of environmental bisphenols and sex hormones [23, 24, 34], several potential confounding variables, including age, race, poverty income ratio (PIR), body mass index, smoking status, urinary creatinine, time of sample collection, and the season of sample collection, were considered covariates. The classification of BMI and smoking status has been described in detail elsewhere [34]. Considering urinary dilution, unadjusted bisphenol concentrations were used as the predictor variable and adjusted for urinary creatinine in the model [35]. The LOD for urinary creatinine was 0.1 mg/dL. Since diurnal variations in sex hormones were observed, the time of sample collection was also adjusted (classification: morning, afternoon and evening) [36]. Moreover, the sample collection season was recorded as a six-month time period, and participants were randomly divided into two categories to measure sex hormones from November 1st through April 30th or May 1st through October 31st.

### Statistical analyses

The continuous variables were expressed as the median and interquartile range (IQR: 25th–75th percentile) or mean ± standard deviation (SD). The categorical variables were expressed as frequencies (percentages). The Student’s t test (normal distribution), Mann–Whitney U test (nonnormal distribution) and chi-square test were performed to compare the differences among different BPA groups. The geometric mean [GM, 95% confidence intervals (CI)] was also calculated because the distribution of bisphenol levels and sex hormones was not normal. Given the nonmonotonic association between BPA exposure and disease outcomes [37], urinary BPA was categorized into quartiles and modeled to allow for nonlinear relationships. The same statistical management was performed for BPF and BPS. For BPF, quartile 1 and quartile 2 were merged into one category because of the low detection rate of 57.14% in total.

To evaluate the association between bisphenols (BPA, BPS, and BPF) and sex hormones, two multivariable regression model analyses, including a crude model (model 1) and model 2 (age, race, PIR, BMI, smoking status, urinary creatinine, time of sample collection, and six-month time period were adjusted) were performed. Linear trend tests were also performed by assigning the median value of each bisphenol category (BPA, BPS, and BPF) in the regression models. Finally, stratified and interaction analyses to test the heterogeneity across subgroups were performed.

Software packages R (http://www.R-project.org, The R Foundation) and Empower (www.empowerstats.com) were used for conducting all data analyses. A two-tailed *p*-value < 0.05 was considered statistically significant.

## Results

A total of 2367 subjects with an average age of 48.91 ± 17.64 years were included in the BPA group, while 1575 participants with an average age of 49.15 ± 17.59 years were included in the BPS/BPF group (Table 1). The mean BMI was 28.80 ± 6.17 kg/m^2^ and 28.91 ± 6.12 kg/m^2^ in the two groups, and over 80% of them were defined as overweight/obese. The median urinary creatinine level was 130.00 mg/dL in the BPA group and 130.50 mg/dL in the BPS/BPF group. Most participants were non-Hispanic white and who donated blood samples in the daytime in both groups. In the BPA group, approximately 17% of participants were reported to be current smokers. When stratified by BPA groups (quartile), the lowest BPA concentration group was slightly older, had a race other than White, Black, or Hispanic, with a normal BMI and the lowest urinary creatinine levels (Supplementary Table 1). The detection frequencies were 96.62, 92.32, and 57.14% for BPA, BPS, and BPF, respectively. The geometric means were 1.40 ng/mL for BPA, followed by 0.55 ng/mL for BPS and 0.44 ng/mL for BPF. Serum TT, E2, and SHBG were detected in 99.00, 99.74, and 100% of the overall samples, respectively. (Supplementary Table 2).

**Table 1:** Population characteristics of study population in the 2011–2016 continuous NHANES

Characteristic	BPA (NHANES 2011-2016)	BPS and BPF (NHANES2013-2016)
Number	2367	1575
Age	48.91±17.64	49.15±17.59
BMI (kg/m^2^)	28.80±6.17	28.91±6.12
Urinary creatinine [mg/dL, median (IQR)]	130.00 (80.00-194.00)	130.50 (81.00-198.00)
% Race		
Mexican American	13.39%	15.37%
Other Hispanic	10.18%	10.48%
Non-Hispanic White	37.52%	38.03%
Non-Hispanic Black	22.48%	20.89%
Other Race	16.43%	15.24%
% PIR		
≤1.3	31.48%	31.18%
1.3-3.5	36.15%	36.23%
>3.5	32.37%	32.59%
% Smoking status		
never	48.48%	48.22%
former	34.56%	35.05%
current	16.96%	16.73%
% Time of venipuncture		
morning	49.39%	49.71%
afternoon	35.74%	35.37%
evening	14.87%	14.92%
% BMI		
Normal (<25kg/m^2^)	48.48%	26.19%
Overweight (25–29.9 kg/m^2^)	34.56%	38.96
Obesity (>=30 kg/m^2^)	16.96%	34.85%
Six-month time period		
November 1 through April 30	48.92%	49.02%
May 1 through October 31	51.08%	50.98%

Mean +/- SD for continuous variables: *P* value was calculated by weighted t-test

% for Categorical variables: *P* value was calculated by weighted chi-square test

*BMI* Body Mass Index

In order to evaluate the association between bisphenols and serum sex hormones concentrations (Table 2), the association of BPA and its analogs with TT in the crude model was first evaluated. Our results showed that significant associations were not observed between BPA or BPA analogs (BPS, BPF) and TT when bisphenols were treated as both continuous and categorical variables. (all *P* > 0.05, *P* for trend > 0.05). Next, a fully adjusted model was applied, which revealed no significant associations, except a positive association between BPF and TT. A per unit increase in BPF was associated with a 0.575 ng/dL higher TT (95% CI: 0.047, 1.103, *P* = 0.033). Then, the association of BPA or its analogs (BPS, BPF) with E_2_ were evaluated which revealed that the associations between bisphenols (BPA, BPS, and BPF) and E_2_ were also not statistically significant in non-adjusted and fully adjusted models. (all *P* > 0.05, *P* for trend > 0.05). Finally, for the association between BPA or BPA analogs (BPS, BPF) and SHBG, a positive association between BPA and SHBG was found (Model 2, β = 0.364, 95% CI: 0.158, 0.571; *P* = 0.001) after adjusting for all confounders. When BPA was treated as a categorical variable (quartiles), participants in quartile 4 of the BPA group had 4.072 nmol/L higher levels of SHBG than those in quartile 1 (Model 2, β = 4.072, 95% CI: 0.746, 7.397, *P* = 0.017; P for trend =0.005). Additionally, a higher BPS level was associated with higher SHBG levels in non-adjusted and fully adjusted models. (Model 1, β = 0.276, 95% CI: 0.068, 0.484, *P* = 0.009; Model 2, β = 0.250, 95% CI: 0.071, 0.429, *P* = 0.006) However, no statistically significant associations between BPF and SHBG were observed.

**Table 2:** Association between Bisphenols and sex hormones (Total Testosterone, Estradiol, Sex hormone-binding globulin) among the US males in NHANES 2011-2016

Bisphenols	Total Testosterone(ng/dL) β(95%CI)		Estradiol (pg/ml) β(95%CI)		Sex hormone-binding globulin (SHBG) (nmol/L) β(95%CI)	
	Model 1	Model 2	Model 1	Model 2	Model 1	Model 2
BPA (Continuous)	-0.156 (-1.248, 0.936) 0.779	0.611 (-0.805, 2.026) 0.398	-0.009 (-0.078, 0.061) 0.807	-0.039 (-0.143, 0.064) 0.456	0.135 (-0.024, 0.294) 0.096	**0.364 (0.158, 0.571) 0.001**
BPA (Quartiles)						
Q1	0	0	0	0	0	0
Q2	-6.109 (-26.68, 14.46) 0.561	-6.314 (-25.840, 13.213) 0.526	-1.430 (-2.835, -0.025) 0.046	-1.534 (-2.948, -0.120) 0.034	-3.933 (-7.329, -0.537) 0.024	0.746 (-2.209, 3.699) 0.621
Q3	-28.229 (-48.657, -7.801) 0.007	-23.175 (-43.634, -2.715) 0.027	-1.129 (-2.529, 0.272) 0.114	-1.667 (-3.154, -0.180) 0.028	-5.136 (-8.482, -1.791) 0.003	-1.784 (-4.856, 1.289) 0.255
Q4	-15.770 (-36.341, 4.801) 0.1335	-6.709 (-29.146, 15.729) 0.558	0.023 (-1.390, 1.436) 0.975	-0.876 (-2.499, 0.747) 0.290	0.228 (-3.148, 3.604) 0.895	**4.072 (0.746, 7.397) 0.017**
P for trend	0.168	0.924	0.369	0.938	0.252	**0.005**
BPS (Continuous)	-0.165 (-1.511, 1.181) 0.810	0.079 (-1.162, 1.321) 0.900	-0.011 (-0.085, 0.064) 0.774	-0.018 (-0.091, 0.056) 0.635	**0.276 (0.068, 0.484) 0.009**	**0.250 (0.071, 0.429) 0.006**
BPS (Quartiles)						
Q1	0	0	0	0	0	0
Q2	-27.161 (-54.670, 0.377) 0.053	-17.705 (-43.628, 8.218) 0.181	0.706 (-0.829, 2.241) 0.366	0.306 (-1.236, 1.849) 0.697	-4.067 (-7.821, -0.313) 0.034	-2.195 (-5.465, 1.074) 0.188
Q3	11.620 (-16.537, 39.776) 0.419	20.794 (-6.427, 48.016) 0.135	2.067 (0.496, 3.636) 0.011	1.738 (0.118, 3.357) 0.036	-1.482 (-5.312, 2.349) 0.448	1.333 (-2.088, 4.754) 0.445
Q4	-11.482 (-39.870, 16.906) 0.428	-8.295 (-36.676, 20.085) 0.567	0.503 (-1.079, 2.086) 0.533	0.140 (-1.548, 1.827) 0.871	-4.017 (-7.868, -0.165) 0.041	-1.678 (-5.254, 1.897) 0.358
P for trend	0.963	0.775	0.856	0.592	0.222	0.549
BPF (Continuous)	0.078 (-0.494, 0.649) 0.790	**0.575 (0.047, 1.103) 0.033**	0.017 (-0.015, 0.049) 0.295	0.010 (-0.022, 0.042) 0.542	0.008 (-0.066, 0.083) 0.825	0.025 (-0.039, 0.089) 0.444
BPF (Quartiles)						
Q1+2	0	0	0	0	0	0
Q3	-8.203 (-30.453, 14.047) 0.470	-13.633 (-34.227, 6.963) 0.195	-0.278 (-1.518, 0.961) 0.660	-0.372 (-1.599, 0.855) 0.553	-0.941 (-3.914, 2.032) 0.535	-2.453 (-4.997, 0.090) 0.059
Q4	11.352 (-10.122, 32.825) 0.300	18.062 (-2.399, 38.522) 0.084	1.446 (0.255, 2.637) 0.017	1.172 (-0.043, 2.384) 0.059	-1.266 (-4.153, 1.622) 0.390	-1.659 (-4.188, 0.870) 0.199
P for trend	0.451	0.223	0.634	0.468	0.527	0.058

Model 1: crude model

Model 2: adjusted for age, race, BMI, poverty income ratio (PIR), smoking status, urinary creatinine, and time of sample collection, six-month time period.

*95%CI* 95% Confidence interval

In order to evaluate the association between bisphenols exposures and calculated FT (Supplementary Table 3) as well as TT/E_2_ (Supplementary Table 4), _._it was demonstrated that there were no significant associations between bisphenols (BPA, BPS, and BPF) and calculated FT in the crude model. (all *P* > 0.05) Then, a fully adjusted model was applied which demonstrated that BPA and BPS were negatively associated with FT (nmol/L) (Model 2, β = − 0.01%, *P* = 0.0211; Model 2, β = − 0.01%, *P* = 0.0258, respectively), while BPF was positively associated with FT (Model 2, β = 0. 0029%, *P* = 0.0028). For TT/E_2_, there were no significant associations between BPA, BPS, and TT/E_2_ when treated with BPA and BPS in either the continuous or categorical variables. However, a per unit increase in BPS was significantly associated with a 0.0001 increase in TT/E_2_ (*P* = 0.0492).

To examine the potential modifying effect of BMI on the associations between bisphenols exposures and hormone concentrations, the association between bisphenols and TT among different BMI groups was evaluated. In stratified analyses, it was found that associations were stronger between BPF and TT among obese men (Table 3, *P* for trend = 0.009; *P* for interaction = 0.027). Then, a fully adjusted model was applied which demonstrated that for men with obesity, compared with participants at quartile 2, those at quartile 3 had 39.027 ng/dL lower TT, and those at quartile 4 had 19.215 ng/dL higher TT (95% CI: − 80.50, − 27.86, *P* = 0.017; 95% CI: − 22.28, 40.01, *P* = 0.225, respectively). Next, for the association between bisphenols exposures and E_2_, the interaction tests reported a significant effect of BMI on the association of BPS with E_2_. Furthermore, the interaction test and stratified analysis also indicated that the association was stronger between BPA and SHBG in men with normal weight when stratified by BMI in a fully adjusted model (*P* for interaction = 0.023). Compared to those in quartile 1, those in quartile 4 had a 9.217 nmol/L higher SHBG (95% CI: 1.753, 16.68; *P* for trend = 0.018). (Table 3) Finally, supplementary Table 5 demonstrates that BMI might be a potential effector on the associations of BPA and FT, and the negative association was stronger in participants with normal BMI.

**Table 3:** Stratified analyses for the association between Bisphenols and sex hormones among different BMI groups in NHANES 2011-2016^a^

Bisphenols	Total Testosterone(ng/dL) β(95%CI)			Estrodiol pg/mL β(95%CI)			sex hormone-binding globulin (SHBG) (nmol/L) β(95%CI)		
BMI	BPA	BPS	BPF^b^	BPA	BPS	BPF^b^	BPA	BPS	BPF^b^
**Normal (BMI<25 kg/m**^**2**^**)**									
Q1	0	0	-	0	0	-	0	0	
Q2	-15.301 (-56.008, 25.406)	-42.339 (-97.7169, 13.039)	0	-0.300 (-2.852, 2.252)	-0.018 (-2.800, 2.764)	0	5.700 (-0.363, 11.763)	-4.155 (-10.778, 2.467)	0
Q3	-44.446 (-90.232, 1.340)	-1.444 (-59.518, 56.629)	19.930 (-26.249, 66.109)	-1.387 (-4.369, 1.595)	-0.406 (-3.322, 2.509)	-0.748 (-3.081, 1.584)	-1.159 (-8.042, 5.724)	-1.604 (-8.544, 5.335)	1.097 (-4.300, 6.495)
Q4	-20.640 (-72.017, 30.736)	-20.426 (-79.364, 38.512)	2.424 (-45.380, 50.229)	-2.128 (-5.400, 1.144)	-0.429 (-3.399, 2.540)	-0.187 (-2.581, 2.207)	9.217 (1.753, 16.681)	-6.470 (-13.603, 0.663)	-0.245 (-5.895, 5.405)
P for trend	0.705	0.980	0.446	0.208	0.758	0.330	0.018	0.125	0.765
**Overweight (BMI 25–29.9 kg/m**^**2**^**)**									
Q1	0	0	-	0	0	-	0	0	
Q2	-21.057 (-52.519, 10.405)	-20.682 (-61.043, 19.680)	0	-1.212 (-3.364, 0.941)	-2.157 (-4.487, 0.173)	0	2.254 (-2.296, 6.805)	-0.121 (-5.177, 4.934)	0
Q3	-44.487 (-77.418, -11.555)	-3.530 (-47.245, 40.185)	-14.431 (-46.821, 17.960) 0.383	-2.627 (-4.878, -0.376)	-1.040 (-3.561, 1.480)	0.071 (-1.792, 1.934)	-0.136 (-4.859, 4.587)	2.647 (-2.736, 8.031)	-2.164 (-6.091, 1.763)
Q4	-18.700 (-55.833, 18.432)	-26.164 (-71.089, 18.762)	21.759 (-10.731, 54.249) 0.190	-0.572 (-3.146, 2.002)	-2.160 (-4.739, 0.419)	1.937 (0.073, 3.801)	3.933 (-1.373, 9.239)	2.236 (-3.249, 7.720)	0.664 (-3.305, 4.634)
P for trend	0.754	0.377	0.651	0.875	0.378	0.684	0.203	0.359	0.419
**Obesity (BMI≥30 kg/m**^**2**^**)**									
Q1	0	0	-	0	0	-		0	
Q2	11.538 (-19.286, 42.361)	6.812 (-35.172, 48.796)	0	-2.901 (-5.562, -0.239)	4.064 (1.187, 6.940)	0	0	-2.416 (-8.132, 3.299)	0
Q3	6.8150 (-23.995, 37.625)	59.052 (16.075, 102.029)	-39.027 (-70.978, -7.077)	-1.056 (-3.748, 1.637)	6.394 (3.449, 9.340)	-0.674 (-2.903, 1.554)	-4.360 (-9.545, 0.825)	2.348 (-3.558, 8.254)	-5.074 (-9.395, -0.753)
Q4	13.431 (-19.277, 46.138)	14.184 (-31.819, 60.188)	19.215 (-11.780, 50.210)	-1.020 (-3.848, 1.807)	3.289 (0.136, 6.442)	1.068 (-1.089, 3.225)	-2.813 (-8.011, 2.386)	-1.824 (-8.211, 4.564)	-5.202 (-9.372, -1.031)
P for trend	0.558	0.897	0.009	0.787	0.855	0.413	0.610	0.655	0.019
P for interaction	0.249	0.288	0.027	0.227	0.007	0.796	0.023	0.732	0.101

*95%CI* 95% Confidence interval

^a^adjusted for adjusted for age, race, poverty income ratio (PIR), smoking status, urinary creatinine, time of sample collection, and six-month time period

^b^for BPF, Q1 and Q2 were merged into Q2, Q3 and Q4 were Q3 and Q4, respectively

## Discussion

This study revealed that higher urinary BPF concentrations were significantly associated with increased TT after adjustment for all confounders. Furthermore, the association was stronger among men with obesity. Additionally, both urinary BPA and BPS concentrations were found to be positively associated with SHBG levels.

Recently, several studies have investigated the associations between BPA exposure and serum sex hormones, but the findings are contradictory. Our finding showed that there was no significant association between BPA and TT or E_2_ in U.S. adult men, which was consistent with previous studies [24, 38]; and Mendiola et al. included 360 U.S. fertile men with a median PSA concentration of 1.7 ng/mL^38^ (1.40 ng/mL in our study). Furthermore, consistent results have also been reported elsewhere across different adult populations (i.e. China, Spanish) [27, 39]. In contrast, both Galloway et al. [25] and Lassen et al. [26] demonstrated that there was a significantly positive association between BPA and TT concentrations from a prospective cohort of young Danish men and Italian adult men, respectively. The median BPA concentration in both studies was more than twofold (3.25 ng/mL and 3.5 ng/mL) than that in our study. In conclusion, these divergent results might be associated with geographic disparities and dissimilarities in exposure levels. Interestingly, inconsistent with previous findings [23, 40], our study reported a positive association between BPF and TT in adult men, which might be explained by the developmental stage of the exposed individual. Our results also indicated that a higher BPA concentration was associated with increased SHBG levels and decreased FT levels, and the positive association between BPA and SHBG has been reported in another study [38]. Currently, SHBG has been identified as a disease risk biomarker [4]; its level is inversely associated with metabolic syndrome and type 2 diabetes, whereas it is positively associated with HDL-cholesterol concentration [41–44]. Regarding FT, it has also been found to be associated with bone health, frailty, and other clinical endpoints [45, 46]. And a previous study reported that determining the FT level avoids under- and over-diagnosis of male hypogonadism and facilitates adequate prescription of hormonal replacement therapy [47]. Therefore, the variations in SHBG and FT concentrations in serum can not only be associated with adverse health outcomes but also impact the sex hormones’ utility as sensitive biomarkers for several clinical outcomes.

The androgenic effect of BPA has been reported elsewhere in some experimental and clinical studies. The possible mechanism for BPA action could be a reduction in aromatase activity due to BPA, which decreased testosterone conversion to E_2_ and increased the TT level [48, 49]. Furthermore, the metabolism of BPA is catalyzed through uridine diphosphate-glucuronosyl transferase (UGT) in the liver and intestine, which produces most of the urinary metabolite BPA-glucuronide [50]. Androgens have been found to reduce the level of UGT activity and transcription [51], which could lead to an increased serum BPA concentration under high androgenic conditions. However, whether BPF acting as a BPA analog might possess similar effects is still unknow. The plausible explanation for the androgenic activity of BPF could be that BPF increased the testicular weight [52] and the cumulative BPF effect increased the Cowper’s gland’s weight [53], showing possible androgenic activity. However, existing evidence could not fully explain the androgenic activity of BPF. To date, the regulation of SHBG has not been fully investigated. In humans, androgen action suppresses the serum SHBG concentration, while estrogen action increases it. Mendiola et al. proposed that the positive association between BPA and SHBG levels could be a direct effect of the estrogenic action of BPA. In addition, BPA might reduce androgenic effects through estrogen receptor-mediated reduction in steroid production [38]. However, associations between BPA analogs (BPS, BPF) and SHBG levels have not been examined. Hence, future studies should be conducted to provide enough evidence to illustrate the role of BPA and its analogs in altering sex hormone levels.

Based on our knowledge, this is the first study to explore the association between urinary concentrations of bisphenol analogs (and not only BPA) and sex hormones in noninstitutionalized adult men using the NHANES database. Our results suggested that BPA substitutes (BPS and BPF) might also have endocrine-disrupting features, and the epidemiologic evidence in our study combined with previous experimental studies suggested that they might not be potentially safe substitutes for BPA. Although our study detected no significant association between BPA and TT, further study should concentrate on the mechanism by which different BPA concentrations may have different effects on TT.

Our study still has some limitations. First, because of the cross-sectional study design, it could not establish causality and could not indicate the impact of long-term exposure. Second, bisphenol exposure was only assessed in a single-spot urine sample. This could result in exposure misclassification and would not establish longitudinal exposure to bisphenol compounds. Previous studies have reported poor reproducibility of BPA and its analogs in spot urine samples through intraclass correlation coefficient estimates (ICC < 0.4) [54–56]. Therefore, repeated urinary specimens are required to characterize bisphenol exposure levels and reduce exposure misclassification. Furthermore, unidentified or unmeasured confounders, like co-exposure to other environmental chemicals, such as phthalate, and dietary patterns related to sex hormones cannot be excluded because the main source of BPA is diet. Finally, although we excluded participants taking sex hormone medications, individuals using other products that could alter sex hormones, like Rogaine and aromatase inhibitors were not excluded.

## Conclusion

In conclusion, this study revealed that BPF was positively associated with TT. It also found that higher BPA and BPS were associated with increased levels of SHBG. Additionally, this study might have potential reproductive health-related impacts, where we demonstrated that BPS and BPF exposure could also have endocrine-disrupting abilities and alter sex hormone levels. This suggests that BPF and BPS might not be safe substitutes for BPA. Further investigation should focus on the endocrine disruption potencies and the mechanisms of BPS and BPF.

## Supplementary Information

**Additional file 1: Supplementary Figure 1.** Participant selection flowchart.

**Additional file 2: Supplementary Table 1.** Population Characteristics of participants stratified by BPA in the 2011–2016 continuous NHANES.

**Additional file 3: Supplementary Table 2.** Distribution of selected Bisphenols and sex hormones in study population, NHANES, USA.

**Additional file 4: Supplementary Table 3.** Association between Bisphenols and free testosterone among the US males in NHANES 2011–2016.

**Additional file 5: Supplementary Table 4.** Association between Bisphenols and Testosterone/estradiol ratio among the US males in NHANES 2011–2016.

**Additional file 6: Supplementary Table 5.** Stratified Analyses for the association between Bisphenols and free testosterone and Testosterone/estradiol ratio among the US males in NHANES 2011–2016*.

## Data Availability

The data sets generated and/or analyzed during the current study are available from the NHANES repository, https://www.cdc.gov/nchs/nhanes/.

## Abbreviations

TT: Total testosterone
E_2_: Estradiol
SHBG: Sex hormone-binding globulin
EDCs: Endocrine disrupting chemicals
BPA: Bisphenol A
BPS: Bisphenol S
BPF: Bisphenol F
LOD: Lower limit of detection
