# Supplementary figures and images for: Associations between urinary concentrations of bisphenols and serum concentrations of sex hormones among US. Males
Source: Environ Health. 2022 Dec 22;21:135. doi: 10.1186/s12940-022-00949-6 (PMC9773582; doi:10.1186/s12940-022-00949-6)

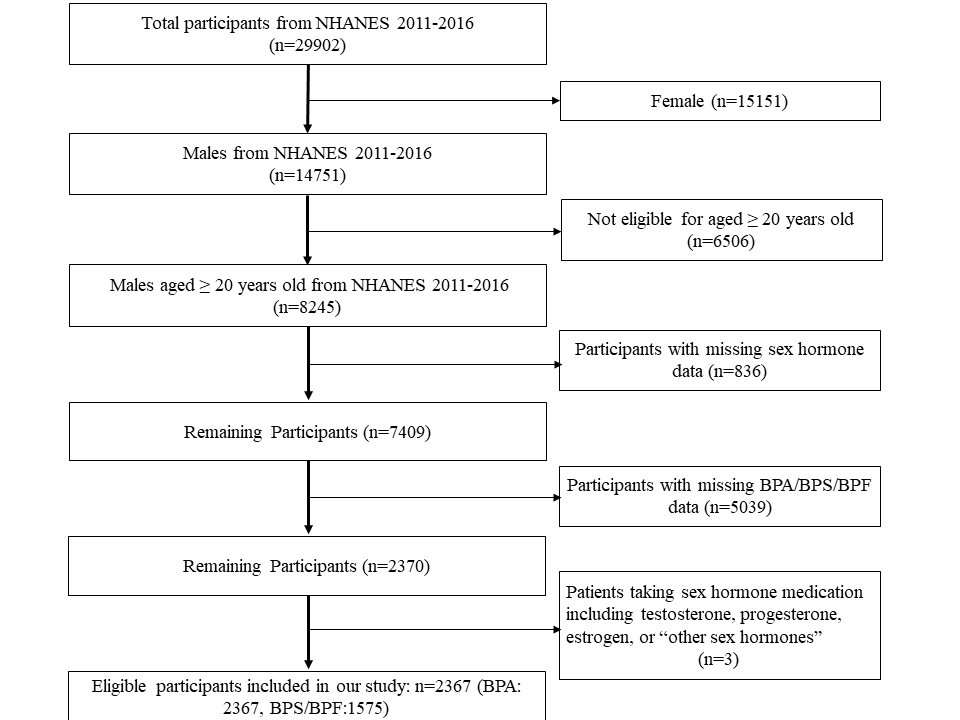

Supplement: Supplementary file 1 — Additional file 1: Supplementary Figure 1. Participant selection flowchart. [file 12940_2022_949_MOESM1_ESM.tif]
