# Supplementary material for: Associations between urinary concentrations of bisphenols and serum concentrations of sex hormones among US. Males
Source: Environ Health. 2022 Dec 22;21:135. doi: 10.1186/s12940-022-00949-6 (PMC9773582; doi:10.1186/s12940-022-00949-6)
Supplement: Supplementary file 2 — Additional file 2: Supplementary Table 1. Population Characteristics of participants stratified by BPA in the 2011–2016 continuous NHANES. [file 12940_2022_949_MOESM2_ESM.docx]

**Supplementary Table 1：Population Characteristics of participants stratified by BPA in the 2011–2016 continuous NHANES**

| Characteristic | Q1 | Q2 | Q3 | Q4 | P value |
| --- | --- | --- | --- | --- | --- |
| Number | 576 | 591 | 597 | 603 |  |
| Age | 51.26 (17.13) | 48.52 (17.64) | 48.20 (17.47) | 47.74 (18.10) | <0.01 |
| BMI (kg/m^2^) | 28.04 ± 5.66 | 28.49 ± 6.09 | 29.20 ± 5.80 | 29.42 ± 4.95 | <0.001 |
| Urinary creatinine [mg/dL, median (IQR)] | 71.00 (40.00-108.00) | 118.00 (81.25-157.00) | 152.00 (106.00-210.00) | 192.00 (140.00-258.50) | <0.001 |
| % Race |  |  |  |  | <0.001 |
| Mexican American | 12.67% | 13.54% | 15.58% | 11.77% |  |
| Other Hispanic | 9.20% | 11.17% | 9.72% | 10.61% |  |
| Non-Hispanic White | 38.37% | 39.26% | 35.51% | 36.98% |  |
| Non-Hispanic Black | 14.24% | 19.12% | 25.29% | 30.85% |  |
| Other Race | 25.52% | 16.92% | 13.90% | 9.78% |  |
| % PIR |  |  |  |  | 0.002 |
| ≤1.3 | 29.87% | 29.79% | 30.56% | 35.58% |  |
| 1.3-3.5 | 33.46% | 36.05% | 35.37% | 39.60% |  |
| >3.5 | 36.67% | 34.16% | 34.07% | 24.82% |  |
| % Smoking status |  |  |  |  | 0.237 |
| never | 49.83% | 48.81% | 49.58% | 45.76% |  |
| former | 34.55% | 35.08% | 35.01% | 33.61% |  |
| current | 15.62% | 16.10% | 15.41% | 20.63% |  |
| % Time of venipuncture |  |  |  |  | 0.004 |
| morning | 49.48% | 51.44% | 50.42% | 46.27% |  |
| afternoon | 38.54% | 33.67% | 36.85% | 34.00% |  |
| evening | 11.98% | 14.89% | 12.73% | 19.73% |  |
| % BMI |  |  |  |  | 0.001 |
| Normal (<25kg/m^2^) | 32.11% | 28.35% | 23.44% | 27.10% |  |
| Overweight (25–29.9 kg/m^2^) | 39.65% | 39.69% | 39.46% | 34.51% |  |
| Obesity (>=30 kg/m^2^) | 28.25% | 31.96% | 37.10% | 38.38% |  |
| Six-month time period |  |  |  |  | 0.026 |
| November 1 through April 30 | 53.30% | 50.08% | 44.72% | 47.76% |  |
| May 1 through October 31 | 46.70% | 49.92% | 55.28% | 52.24% |  |

Mean +/- SD for continuous variables: P value was calculated by weighted t-test.

% for Categorical variables: P value was calculated by weighted chi-square test.
