# Supplementary material for: Associations between urinary concentrations of bisphenols and serum concentrations of sex hormones among US. Males
Source: Environ Health. 2022 Dec 22;21:135. doi: 10.1186/s12940-022-00949-6 (PMC9773582; doi:10.1186/s12940-022-00949-6)
Supplement: Supplementary file 3 — Additional file 3: Supplementary Table 2. Distribution of selected Bisphenols and sex hormones in study population, NHANES, USA. [file 12940_2022_949_MOESM3_ESM.docx]

| **Bisphenols and sex hormones** | **Detection rate** | **GM (95%CI)** | **Mean** | **Percentile** | | | | |
| --- | --- | --- | --- | --- | --- | --- | --- | --- |
|  |  |  |  | **25th percentile** | | **50th percentile** | **75th percentile** | |
| **Bisphenols** |  |  |  |  |  | |  | |
| Bisphenol A | 96.62% | 1.40 (1.34 1.46) | 2.93 | 0.70 | | 1.40 | | 2.80 |
| Bisphenol S | 92.32% | 0.55 (0.51 0.58) | 1.63 | 0.20 | | 0.50 | | 1.10 |
| Bisphenol F | 57.14% | 0.44 (0.41 0.48) | 2.67 | 0.14 | | 0.30 | | 0.90 |
| **Sex hormones** |  |  |  |  | |  | |  |
| TT, ng/dl | 99.00% | 367.08 (358.93 375.43) | 412.26 | 286.25 | | 382.00 | | 506.00 |
| E2, pg/ml | 99.74% | 23.42 (22.94 23.91) | 25.40 | 18.60 | | 23.90 | | 30.30 |
| SHBG, nmol/L | 100.00% | 39.45 (38.39 40.54) | 45.25 | 28.05 | | 39.53 | | 55.72 |
| Calculated free T, nmol/L | - | 0.02 (0.02 0.02) | 0.02 | 0.01 | | 0.02 | | 0.02 |
| Testosterone/Estradiol | - | 0.05 (0.04 0.05) | 0.05 | 0.04 | | 0.05 | | 0.06 |

**Supplementary Table 2. Distribution of selected Bisphenols and sex hormones in study population, NHANES, USA.**
