# Supplementary material for: Associations between urinary concentrations of bisphenols and serum concentrations of sex hormones among US. Males
Source: Environ Health. 2022 Dec 22;21:135. doi: 10.1186/s12940-022-00949-6 (PMC9773582; doi:10.1186/s12940-022-00949-6)
Supplement: Supplementary file 4 — Additional file 4: Supplementary Table 3. Association between Bisphenols and free testosterone among the US males in NHANES 2011–2016. [file 12940_2022_949_MOESM4_ESM.docx]

**Supplementary Table 3: Association between Bisphenols and free testosterone among the US males in NHANES 2011-2016.**

| **Bisphenols** | Free testosterone (nmol/L)  β(95%CI) | |
| --- | --- | --- |
|  | Model 1 | Model 2 |
| BPA (continuous) | -0.0000 (-0.0001, 0.0000) 0.0788 | **-0.0001 (-0.0001, -0.0000) 0.0211** |
| BPA (Quartiles) |  |  |
| Q1 | 0 | 0 |
| Q2 | 0.0007 (-0.0003, 0.0017) 0.1939 | -0.0002 (-0.0011, 0.0007) 0.7002 |
| Q3 | 0.0005 (-0.0005, 0.0015) 0.3657 | -0.0001 (-0.0010, 0.0008) 0.8081 |
| Q4 | -0.0001 (-0.0011, 0.0009) 0.8567 | -0.0006 (-0.0016, 0.0004) 0.2389 |
| P for trend | 0.4437 | 0.2207 |
| BPS (continuous) | -0.0001 (-0.0001, 0.0000) 0.0567 | **-0.0001 (-0.0001, -0.0000) 0.0258** |
| BPS (Quartiles) |  |  |
| Q1 | 0 | 0 |
| Q2 | -0.0002 (-0.0014, 0.0009) 0.6959 | -0.0005 (-0.0015, 0.0005) 0.3040 |
| Q3 | 0.0009 (-0.0003, 0.0020) 0.1413 | 0.0002 (-0.0008, 0.0013) 0.6699 |
| Q4 | 0.0004 (-0.0007, 0.0016) 0.4738 | -0.0005 (-0.0016, 0.0005) 0.3381 |
| P for trend | 0.3375 | 0.4441 |
| BPF (continuous) | 0.0000 (-0.0000, 0.0000) 0.3847 | **0.0000 (0.0000, 0.0000) 0.0028^*^** |
| BPF (Quartiles) |  |  |
| Q1+2 | 0 | 0 |
| Q3 | -0.0002 (-0.0010, 0.0007) 0.7403 | -0.0001 (-0.0009, 0.0006) 0.7590 |
| Q4 | 0.0006 (-0.0002, 0.0015) 0.1510 | 0.0010 (0.0002, 0.0017) 0.0126 |
| P for trend | 0.7208 | 0.8994 |

95%CI: 95% Confidence interval

Model 1: crude model

Model 2: adjusted for age, race, BMI, poverty income ratio (PIR), smoking status, urinary creatinine, and time of sample collection, six-month time period.

*: 0.0000293 (0.0000101, 0.0000484)
