# Supplementary material for: Associations between urinary concentrations of bisphenols and serum concentrations of sex hormones among US. Males
Source: Environ Health. 2022 Dec 22;21:135. doi: 10.1186/s12940-022-00949-6 (PMC9773582; doi:10.1186/s12940-022-00949-6)
Supplement: Supplementary file 5 — Additional file 5: Supplementary Table 4. Association between Bisphenols and Testosterone/estradiol ratio among the US males in NHANES 2011–2016. [file 12940_2022_949_MOESM5_ESM.docx]

**Supplementary table 4: Association between Bisphenols and Testosterone/estradiol ratio among the US males in NHANES 2011-2016.**

| **Bisphenols** | Testosterone/estradiol ratio  β(95%CI) | |
| --- | --- | --- |
|  | Model 1 | Model 2 |
| BPA (continuous) | 0.0000 (-0.0002, 0.0002) 0.9558 | 0.0002 (-0.0000, 0.0005) 0.1110 |
| BPA (Quartiles) |  |  |
| Q1 | 0 | 0 |
| Q2 | 0.0012 (-0.0025, 0.0049) 0.5218 | 0.0024 (-0.0011, 0.0059) 0.1763 |
| Q3 | -0.0024 (-0.0060, 0.0013) 0.2070 | 0.0006 (-0.0031, 0.0043) 0.7507 |
| Q4 | -0.0006 (-0.0043, 0.0031) 0.7486 | 0.0033 (-0.0007, 0.0073) 0.1023 |
| P for trend | 0.5337 | 0.1776 |
| BPS (continuous) | -0.0000 (-0.0002, 0.0002) 0.9322 | 0.0001 (-0.0001, 0.0002) 0.5305 |
| BPS (Quartiles) |  |  |
| Q1 | 0 | 0 |
| Q2 | -0.0043 (-0.0083, -0.0002) 0.0377 | -0.0019 (-0.0057, 0.0019) 0.3373 |
| Q3 | -0.0008 (-0.0049, 0.0033) 0.7053 | 0.0017 (-0.0023, 0.0057) 0.4058 |
| Q4 | -0.0032 (-0.0073, 0.0010) 0.1350 | -0.0011 (-0.0053, 0.0030) 0.5995 |
| P for trend | 0.5531 | 0.7628 |
| BPF (continuous) | -0.0000 (-0.0001, 0.0001) 0.9964 | **0.0001 (0.0000, 0.0002) 0.0492** |
| BPF (Quartiles) |  |  |
| Q1+2 | 0 | 0 |
| Q3 | -0.0007 (-0.0039, 0.0026) 0.6905 | -0.0008 (-0.0039, 0.0022) 0.5888 |
| Q4 | -0.0014 (-0.0045, 0.0018) 0.3940 | 0.0004 (-0.0026, 0.0034) 0.7772 |
| P for trend | 0.6347 | 0.6380 |

95%CI: 95% Confidence interval

Model 1: crude model

Model 2: adjusted for age, race, BMI, poverty income ratio (PIR), smoking status, urinary creatinine, and time of sample collection, six-month time period.
