# Supplementary material for: Associations between urinary concentrations of bisphenols and serum concentrations of sex hormones among US. Males
Source: Environ Health. 2022 Dec 22;21:135. doi: 10.1186/s12940-022-00949-6 (PMC9773582; doi:10.1186/s12940-022-00949-6)
Supplement: Supplementary file 6 — Additional file 6: Supplementary Table 5. Stratified Analyses for the association between Bisphenols and free testosterone and Testosterone/estradiol ratio among the US males in NHANES 2011–2016*. [file 12940_2022_949_MOESM6_ESM.docx]

**Supplementary Table 5: Stratified Analyses for the association between Bisphenols and free testosterone and Testosterone/estradiol ratio among the US males in NHANES 2011-2016*.**

| Bisphenols | free testosterone (nmol/L)  β(95%CI) | | | **Testosterone/estradiol ratio**  β(95%CI) | | |
| --- | --- | --- | --- | --- | --- | --- |
| BMI | BPA | BPS | BPF^#^ | BPA | BPS | BPF^#^ |
| **Normal (BMI<25 kg/m^2^)** |  |  |  |  | | |
| Q1 | 0 | 0 | - | 0 | 0 | - |
| Q2 | 0.000 (-0.001, 0.002) | -0.001 (-0.003, 0.000) | 0 | 0.004 (-0.003, 0.010) | -0.008 (-0.015, -0.001) | 0 |
| Q3 | -0.001 (-0.002, 0.001) | -0.001 (-0.003, 0.001) | 0.000 (-0.001, 0.002) | -0.002 (-0.010, 0.005) | -0.000 (-0.008, 0.007) | 0.003 (-0.003, 0.009) |
| Q4 | -0.003 (-0.005, -0.001) | 0.000 (-0.002, 0.002) | -0.000 (-0.002, 0.001) | 0.005 (-0.003, 0.014) | -0.006 (-0.014, 0.001) | -0.000 (-0.006, 0.006) |
| P for trend | 0.001 | 0.256 | 0.951 | 0.196 | 0.376 | 0.184 |
| **Overweight (BMI** **25–29.9 kg/m^2^)** |  |  |  |  |  |  |
| Q1 | 0 | 0 | - | 0 | 0 | - |
| Q2 | -0.002 (-0.003, -0.000) | -0.001 (-0.003, 0.001) | 0 | -0.001 (-0.005, 0.003) | 0.003 (-0.002, 0.007) | 0 |
| Q3 | -0.002 (-0.003, -0.000) | -0.001 (-0.003, 0.001) | -0.000 (-0.001, 0.001) | -0.002 (-0.006, 0.002) | 0.002 (-0.003, 0.006) | -0.000 (-0.004, 0.003) |
| Q4 | -0.002 (-0.003, 0.000) | -0.003 (-0.005, -0.001) | 0.000 (-0.001, 0.002) | 0.000 (-0.005, 0.005) | 0.001 (-0.003, 0.006) | -0.000 (-0.003, 0.003) |
| P for trend | 0.468 | 0.004 | 0.753 | 0.775 | 0.829 | 0.903 |
| **Obesity (BMI≥30 kg/m^2^)** |  |  |  |  |  |  |
| Q1 | 0 | 0 | - | 0 | 0 | - |
| Q2 | 0.001 (-0.000, 0.003) | 0.001 (-0.001, 0.002) | 0 | 0.005 (-0.002, 0.013) | -0.003 (-0.012, 0.005) | 0 |
| Q3 | 0.002 (0.000, 0.003) | 0.002 (0.000, 0.004) | -0.000 (-0.001, 0.001) | 0.006 (-0.002, 0.013) | 0.002 (-0.007, 0.011) | -0.003 (-0.010, 0.003) |
| Q4 | 0.001 (-0.000, 0.003) | 0.001 (-0.001, 0.003) | 0.002 (0.001, 0.003) | 0.007 (-0.001, 0.015) | -0.000 (-0.009, 0.009) | 0.002 (-0.004, 0.009) |
| P for trend | 0.414 | 0.796 | 0.556 | 0.219 | 0.765 | 0.036 |
| P for interaction | 0.021 | 0.112 | 0.017 | 0.351 | 0.720 | 0.081 |

95%CI: 95% Confidence interval

*adjusted for adjusted for age, race, poverty income ratio (PIR), smoking status, urinary creatinine, and time of sample collection, six-month time period.

#for BPF, Q1 and Q2 were merged into Q2, Q3 and Q4 were Q3 and Q4, respectively.
